# Supplementary material for: A set of multi-entry identification keys to African frugivorous flies (Diptera, Tephritidae)
Source: Zookeys. 2014 Jul 24;(428):97–108. doi: 10.3897/zookeys.428.7366 (PMC4143993; doi:10.3897/zookeys.428.7366)
Supplement: Supplementary material 4 — Key to Capparimyia [file zookeys-428-097-s004.zip › SF4_ZooKeys_key to Capparimyia/lucid_player/help/help/whnjs.htm]

Lucid3 ver3\_4 Player Help

<p>Your browser does not support frames. We recommend that you update your browser to a version that supports frames.</p><p>To view the Help system without frames click on this<a href="whgdata/whnvp30.htm">hyperlink.</a></p>
